# Supplementary material for: The Effects of TGF-β Signaling on Cancer Cells and Cancer Stem Cells in the Bone Microenvironment
Source: Int J Mol Sci. 2019 Oct 15;20(20):5117. doi: 10.3390/ijms20205117 (PMC6829436; doi:10.3390/ijms20205117)
Supplement: Supplementary file 1 [file ijms-20-05117-s001.pdf]

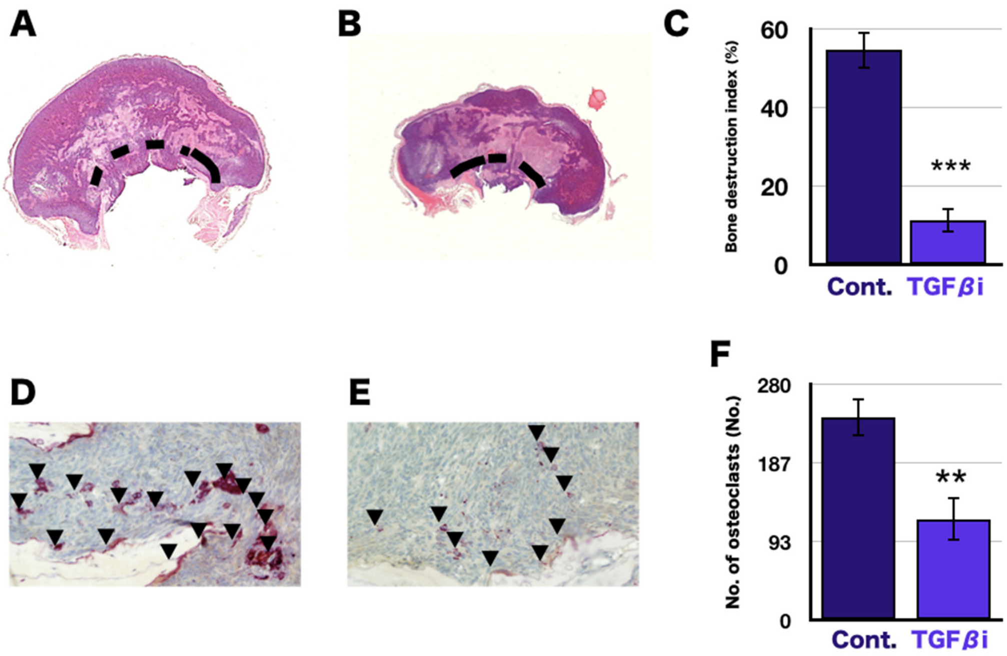

Supplementary Figure S1: The effects of TGF-β signaling on osteoclast induction and osteolysis. (**A**) Length of osteolysis in the cranial bone of the control group. (**B**) Less osteolysis in the of R1- Ki treatment group. (**C**) Quantitative analysis of the degree of osteolysis. (**D**) A large number of osteoclasts was detected by Tartrate-Resistant Acid Phosphatase (TRAP) staining in the control group. Black triangle indicate osteoclast. (**E**) Fewer osteoclasts were detected in the R1- Ki treatment group. Black triangle indicate osteoclast. (**F**) Quantitative analysis of the induction of osteoclasts. **, ***: $p < 0.01$, $p < 0.001$ versus Con and Control groups.

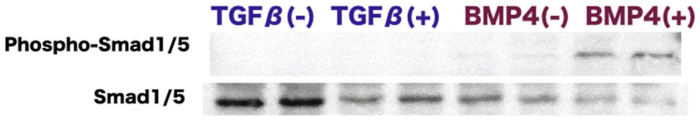

Supplementary Figure S2: Confirmation of the expression of *p*-SMAD1/5 and SMAD1/5. Expression of p-SMAD1/5 and SMAD1/5 was confirmed by the extracts from BMP-4 treated HeLa cells and untreated HeLa cells, respectively.
